# Visible Particle Series Search Algorithm and Its Application in Structural Damage Identification

**DOI:** 10.3390/s22031275

**Published:** 2022-02-08

**Authors:** Pooya Mohebian, Seyed Bahram Beheshti Aval, Mohammad Noori, Naiwei Lu, Wael A. Altabey

**Affiliations:** 1 Faculty of Civil Engineering, K. N. Toosi University of Technology, Tehran 196976-4499, Iran; mohebian@email.kntu.ac.ir (P.M.); beheshti@kntu.ac.ir (S.B.B.A.); 2 Department of Mechanical Engineering, California Polytechnic State University, San Luis Obispo, CA 93405, USA; 3 School of Civil Engineering, Changsha University of Science and Technology, Changsha 410076, China; lunaiwei@csust.edu.cn; 4 International Institute for Urban Systems Engineering, Southeast University, Nanjing 210096, China; 5 Department of Mechanical Engineering, Faculty of Engineering, Alexandria University, Alexandria 21544, Egypt

**Keywords:** structural damage identification, health monitoring, optimization method, meta-heuristic algorithm, visible particle series search

## Abstract

Identifying structural damage is an essential task for ensuring the safety and functionality of civil, mechanical, and aerospace structures. In this study, the structural damage identification scheme is formulated as an optimization problem, and a new meta-heuristic optimization algorithm, called visible particle series search (VPSS), is proposed to tackle that. The proposed VPSS algorithm is inspired by the visibility graph technique, which is a technique used basically to convert a time series into a graph network. In the proposed VPSS algorithm, the population of candidate solutions is regarded as a particle series and is further mapped into a visibility graph network to obtain visible particles. The information captured from the visible particles is then utilized by the algorithm to seek the optimum solution over the search space. The general performance of the proposed VPSS algorithm is first verified on a set of mathematical benchmark functions, and, afterward, its ability to identify structural damage is assessed by conducting various numerical simulations. The results demonstrate the high accuracy, reliability, and computational efficiency of the VPSS algorithm for identifying the location and the extent of damage in structures.

## 1. Introduction

Civil, aerospace, and mechanical structural systems may accumulate some local damage during their operational life as a consequence of different unfavorable conditions, such as excess loads, fatigue, corrosion, high intensity loads, or earthquake. When such damages remain undetected and unrepaired, they can negatively impact the functionality and integrity of the structure and may even lead to structural failure. Accordingly, structural damage identification plays a crucial role in achieving the maintainability, safety, and reliability of structures [1,2,3,4,5,6,7,8,9,10,11,12,13,14,15,16].

Over the past few decades, vibration-based methods have been developed for structural damage identification [17,18,19,20,21,22,23]. The underlying idea behind these methods comes from the fact that modal parameters are linked to physical parameters of the structure. Hence, any modifications in the physical properties due to damage can be detected by evaluating variations in the modal properties [21,22,23,24,25,26].

Mathematically, vibration-based damage identification can be formulated within the framework of an optimization problem. In this manner, the locations and extents of damage are taken as variables of the optimization problem, and the objective function is specified in terms of differences between the measured vibration data and those computed from the finite element model of the structure. An optimization algorithm is then utilized to deal with the problem by minimizing the objective function [27,28,29,30,31,32].

Traditional optimization algorithms require complex gradient calculations and usually get trapped in local optima [33]. Therefore, in recent years, meta-heuristic algorithms have received considerable attention in the field of structural damage identification owing to their simplicity, versatility, and robustness. For instance, Hao and Xia [33] employed the genetic algorithm (GA) to cope with the structural damage detection problem. Mohan et al. [34] adopted the particle swarm optimization (PSO) algorithm for identifying damage in beam and frame structures. Majumdar et al. [35] performed damage identification in truss structures by employing the ant colony optimization (ACO) algorithm. Torkzadeh, Ghiasi, and Noori [36] utilized a particle swarm harmony search (PSH) combined with artificial neural networks and the least-squares support-vector machine to detect damage in truss and frame structures. Ding et al. [37] proposed an artificial bee colony (ABC) algorithm with a hybrid search strategy to address the structural damage identification problem. Seyedpoor et al. [38] applied the differential evolution (DE) algorithm for structural damage identification. Wang, Noori, Altabey, et al. [39] used the particle swarm optimization (PSO) in conjunction with the least-mean-square algorithm for system identification of a hysteretic system. Xu et al. [27] utilized the cuckoo search (CS) algorithm for identifying damage in beam and truss structures. Kaveh and Zolghadr [40] proposed an improved charged system search (CSS) algorithm for damage identification of truss structures. Zhu et al. [41] employed the bird mating optimizer (BMO) for assessing structural damage. Ghannadi, Kourehli, Noori, et al. [42] employed a gray wolf optimizer (GWO) combined with a mode shape expansion scheme to study structural damage detection. Nobahari et al. [43] developed a new optimization algorithm called echolocation search algorithm (ESA) to identify the location and the extent of damage in structures. Fallah et al. [44] applied the crow search algorithm (CSA) for damage severity assessment of large-scale truss structures. Fathi et al. [45] carried out crack detection in plate structures by the extended finite element method and an enhanced vibrating particles system (EVPS). Du et al. [46] adopted the Jaya algorithm to deal with the damage detection problem of truss and frame structures. Dinh-Cong et al. [28] proposed a method for structural damage assessment by using lightning attachment procedure optimization (LAPO). Dinh-Cong et al. [47] presented an optimization-based technique for damage identification in full-scale structures with the aid of an enhanced symbiotic organisms search (ESOS) algorithm and the commercial software SAP2000-OAPI. Mishra et al. [48] examined the effectiveness of the ant lion optimizer (ALO) for solving different damage detection problems. Mishra et al. [49] conducted damage identification of large-scale spatial truss structures by employing teaching–learning-based optimization (TLBO). Beheshti Aval and Mohebian [50] proposed an improved biology migration algorithm (IBMA) to conduct the combined joint and member damage identification of skeletal structures. Chen and Yu [51] proposed a hybrid algorithm combining ALO with an improved Nelder-Mead algorithm for structural damage detection. Ding et al. [52] presented a hybrid optimization algorithm based on the Jaya and tree seeds algorithm (TSA) to accomplish structural damage identification. Beheshti Aval and Mohebian [53] proposed a method for the joint damage identification of frame structures by employing the equilibrium optimizer (EO) algorithm. Tiachacht et al. [54] utilized the slime mold algorithm (SMA) to identify damage in structures. Huang et al. [55] introduced a new damage detection method by using an enhanced moth-flame optimization (EMFO). Ghannadi and Kourehli [56] investigated the application of the multiverse optimizer (MVO) for dealing with the damage identification problem.

Despite an extensive list of meta-heuristic algorithms, some of which were mentioned above, none of them have been specifically developed for structural damage identification, and only an application of those algorithms in this field has been investigated. Furthermore, according to the no free lunch (NFL) theorem [57], there is no optimization algorithm that is superior to other optimization algorithms and capable of solving all optimization problems. In other words, there is always a need to devise new optimization algorithms. With these points in mind, the present study proposes a novel optimization algorithm called visible particle series search (VPSS) to address the structural damage identification problem. The main inspiration for VPSS is based on the visibility graph technique [58], which is adopted from the context of time series analysis. According to the visibility graph technique, a time series is mapped into a graph network from which the inherent characteristics of the time series can be derived and analyzed. In a similar fashion, in the VPSS algorithm, the population of candidate solutions is considered as a particle series and is further converted into a visibility graph network to obtain visible particles associated with each individual. The algorithm then makes use of information provided from the visible particles to effectively update the position of each particle in the search space. In order to examine the general performance of the VPSS algorithm in terms of exploration and exploitation capabilities, it is first applied on a set of 12 mathematical benchmark functions, including unimodal and multi-modal functions. Next, the effectiveness of the proposed method for identifying structural damage is evaluated by using four numerical examples, comprising a 47-bar planar truss, a 54-bar space truss, a two-bay three-story frame, and a television (TV) tower under both noise-free and noisy conditions. For each optimization problem, the results attained by the VPSS algorithm are also compared with those achieved by four other well-known meta-heuristic algorithms, namely PSO [59], DE [60], GWO [61], and LAPO [62].

The rest of this paper is organized as follows. In Section 2, the problem formulation of optimization-based structural damage identification is described. Section 3 introduces the VPSS algorithm and its background inspiration. In Section 4, the general applicability of the proposed algorithm is validated through a set of mathematical benchmark functions. Section 5 investigates the application of the VPSS algorithm in structural damage identification. Finally, the conclusions are presented in Section 6.

## 2. Problem Formulation

Evaluating dynamic characteristics of a structure is an essential part of the structural damage identification process. For the finite element model of the structure, the modal parameters can be determined by addressing the eigenvalue problem specified as follows [37,63]:

(1) $$\left(K-\omega_{j}^{2}M\right)\cdot \Phi_{j}=0,j=1,2,\ldots ,Ndf,$$

where K and M represent the global stiffness and mass matrices, respectively; $\omega_{j}$ is the *j*th natural frequency; $\Phi_{j}$ refers to the *j*th mode shape vector; and *Ndf* is the number of degrees of freedom (NDOFs). 

As a consequence of damage, the stiffness capability of the structure decreases, but its mass characteristic is assumed to remain unchanged. In order to incorporate damage into the finite element formulation of the structure, a damage parameter, $\alpha_{i}$, is considered and is applied to each elemental stiffness matrix. Accordingly, the global stiffness matrix of the structure in the damaged state can be obtained as follows [63]:

(2) $$K_{d}=\sum_{i=1}^{Ne}(1-\alpha_{i})k_{i},$$

where $k_{i}$ stands for the stiffness matrix of the *i*th element in the healthy state; *Ne* denotes the total number of structural elements; and $\alpha_{i}$ implies the damage severity of the *i*th element. The value of $\alpha_{i}$ belongs to the interval [0, 1], where $\alpha_{i}=0$ reflects a perfectly intact state, while $\alpha_{i}=1$ represents a fully damaged state for the *i*th element.

In the context of optimization-based structural damage identification, the aim is to search for a set of damage parameters in such a way that an objective function defined as the differences between the actual and computed vibration data of the structure is minimized. Mathematically, the optimization problem formulation can be stated as follows [3]:

(3) $$\begin{array}{ll} \mathrm{Find} & X=\{\alpha_{1},\alpha_{2},\ldots ,\alpha_{Ne}\}\\ \mathrm{To}\;\mathrm{minimize} & f(X)\\ \mathrm{Subject}\;\mathrm{to} & X^{l}\leq X\leq X^{u} \end{array},$$

where X is the damage variable vector; f(X) is the objective function to be minimized; and $X^{l}$ and $X^{u}$ are the vectors of lower and upper bounds, respectively.

In the present study, the objective function is described according to the natural frequencies and modal assurance criteria (MAC) as follows [63]:

(4) $$f(X)={\sum_{j=1}^{Nf}W_{\omega j}\left(\frac{\omega_{j}^{c}-\omega_{j}^{a}}{\omega_{j}^{a}}\right)}^{2}+\sum_{j=1}^{Nm}W_{\Phi j}\left(1-MAC_{j}\right),$$

in which

(5) $$MAC_{j}=\frac{{\left(\Phi_{j}^{cT}\cdot \Phi_{j}^{a}\right)}^{2}}{{\left\|\Phi_{j}^{c}\right\|}^{2}{\left\|\Phi_{j}^{a}\right\|}^{2}},$$

where $W_{\omega j}$ and $W_{\Phi j}$ are the weight coefficients related to the jth natural frequency and jth *MAC*, respectively, which are considered herein to be unity; $\omega_{j}^{c}$ and $\omega_{j}^{a}$ denote the *j*th computed and actual natural frequencies, respectively; $\Phi_{j}^{c}$ and $\Phi_{j}^{a}$ signify the *j*th computed and actual mode shape vectors, respectively; and *Nf* and *Nm* refer to the number of natural frequencies and mode shapes, respectively.

## 3. Visible Particle Series Search Algorithm

This section intends to introduce a new meta-heuristic optimization algorithm called visible particle series search (VPSS). In the following, first, a background on the visibility graph technique is provided, and then the VPSS algorithm is described in detail.

### 3.1. Background of the Visibility Graph Technique

A time series consists of a set of observational data gathered sequentially in time [64]. In many different areas, such as economics, natural sciences, engineering, etc., data emerge as a time series. For instance, the daily market price of a stock, the hourly air temperature, and the ground motion during an earthquake can be expressed as a time series. One of the inherent aspects of the time series is that adjacent data points are generally related to each other. In order to investigate this relationship and other statistical features of data, time series analysis methods are basically employed [64]. In recent years, graph network approaches have been proposed as efficient tools to analyze time series data. In this fashion, a time series is transformed into an equivalent graph network, which further allows information embedded in the time series to be extracted and characterized [65,66,67]. The visibility graph technique proposed by Lacasa et al. [58] is one of the most widely utilized approaches to map a time series into a graph network. The fundamental concept of this algorithm is presented below.

Consider a time series denoted by $\{(t_{i},s(t_{i})),\;i=1,\ldots ,Ns\},$ containing Ns measured data at successive times. According to the visibility graph method, two arbitrary data points $\left(t_{i},s(t_{i})\right)$ and $\left(t_{j},s(t_{j})\right)$ from the time series are taken to be visible to each other provided that any other data point $\left(t_{k},s(t_{k})\right)$ that lies between them satisfies the following equation [58,68]:

(6) $$s(t_{k})<s(t_{j})+\left(s(t_{i})-s(t_{j})\right)\frac{t_{j}-t_{k}}{t_{j}-t_{i}}.$$

**Figure 1:**
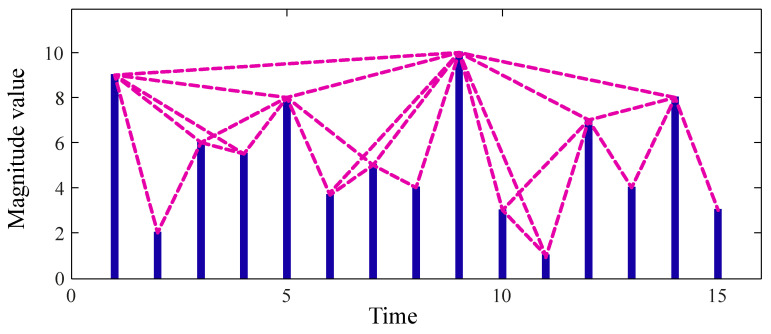
The visibility graph of a time-series.

Figure 1 depicts an example of the visibility graph for a time series. In this figure, each data point of the time series is plotted by a vertical bar, whose height represents its amplitude value. As can be observed, any two visible bars are connected by a straight line that is not intersected by any intermediate bars between them.

### 3.2. The VPSS Algorithm

The VPSS algorithm is a novel population-based meta-heuristic algorithm inspired by the visibility graph theory, described in the previous subsection. In the framework of this algorithm, the population of candidate solutions is first converted into a particle series representation. Each individual in the particle series is analogous to a data point in a time series. Then, the visibility graph network for the particle series is constructed according to the visibility graph theory, and the visible particles associated with each individual are determined and utilized to evolve the population towards the optimum solution. This algorithm needs only the common controlling parameters, including population size and the number of iterations, and does not rely on any algorithm-specific parameter. The basic steps for implementing the VPSS algorithm are outlined as follows.

#### 3.2.1. Step 1: Initialization

The VPSS algorithm starts by randomly generating a population of *Np* candidate solutions within the search space as follows:

(7) $$X_{i}=X^{l}+rand\cdot (X^{u}-X^{l}),i=1,\ldots ,Np,$$

where $X_{i}$ is the initial position vector of the *i*th candidate solution; $X^{l}$ and $X^{u}$ denote the lower and upper bound vectors of the variables, respectively; and rand is a random vector whose components are uniformly distributed within the range of [0, 1]. After initializing the candidate solutions, their fitness function values are also evaluated. For a minimization problem, the fitness function is defined as the inverse of the objective function, i.e., $fit(X_{i})=1/f(X_{i})$, where $fit(X_{i})$ and $f(X_{i})$ represent the fitness function value and the objective function value of the *i*th candidate solution, respectively.

#### 3.2.2. Step 2: Particle Series Construction

**Figure 2:**
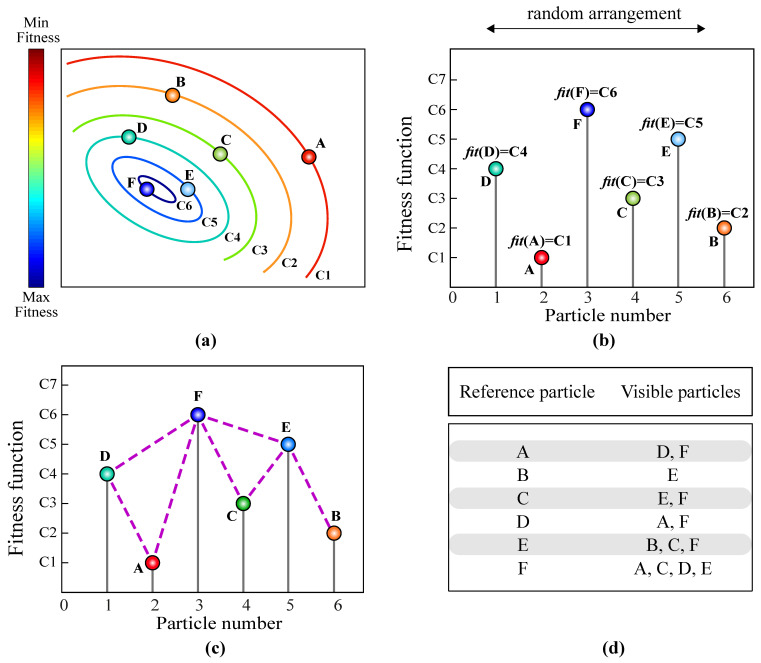
A simple example of the visibility criterion assessment in the VPSS algorithm: (**a**) the population of candidate solutions in the search space; (**b**) converting the population into a particle series representation; (**c**) the corresponding visibility graph for the particle series; (**d**) visible particles associated with each reference particle.

In this stage, the population of the VPSS algorithm is mapped into a particle series representation, denoted by {(*i*, *fit*(**X***_i_*)), *i* = 1, …, *Np*}. The sequence of individuals in the particle series is arranged randomly at each iteration of the algorithm. In addition, particle series amplitude values are considered to be equivalent to the fitness function values of the candidate solutions. As an example, Figure 2a illustrates a population of candidate solutions distributed over the search space. The contour lines provided in this figure indicate the fitness function values for the particles such that $C_{1}<C_{2}<,\ldots ,C_{6}$. Figure 2b presents the particle series representation of the population by considering a random arrangement for the individuals. Obviously, particles with better fitness function values possess a higher height in the graph and vice versa.

#### 3.2.3. Step 3: Visibility Criterion Assessment

In this step, a visibility assessment is carried out to determine the visible particles associated with each individual in the particle series constructed in the previous stage. Two arbitrary individuals $\left(i,fit(X_{i})\right)$ and $\left(j,fit(X_{j})\right)$ from the particle series are considered to be visible to each other if any other particle $\left(k,fit(X_{k})\right)$ between them meets the following criterion:

(8) $$fit(X_{k})<fit(X_{j})+\left(fit(X_{i})-fit(X_{j})\right)\frac{j-k}{j-i},$$

where $fit(X_{i})$,$fit(X_{j})$, and $fit(X_{k})$ are the fitness function values of the *i*th, *j*th, and *k*th particles, respectively.

The corresponding visibility graph of the particle series shown in Figure 2b is indicated in Figure 2c. According to this figure, the visible particles associated with each individual can be recognized. Furthermore, the visible particles associated with each reference particle are shown in Figure 2d.

#### 3.2.4. Step 4: Generation of New Solutions

In this stage, the information captured from the visible particles is utilized to update the position of their corresponding reference particle. Within this context, two updating operators are applied to seek the optimum solution. These updating operators rely on three kinds of visible particles, including the best visible particle, the worst visible particle, and the average of the visible particles.

The first updating operator of the VPSS algorithm consists of two main parts. In the first part, each particle tends to move towards its best visible particle, and, in the second part, attempts to move towards the vector defined by the differences between its best visible particle and the mean position of all its visible particles. This search strategy can be implemented as follows:

(9) $$X_{i}^{new}(t)=X_{i}(t)+rand_{1}\cdot \left(X_{BV,i}(t)-X_{i}(t)\right)+\beta \cdot rand_{2}\cdot \left(X_{BV,i}(t)-\lambda \cdot \;X_{MV,i}(t)\right),$$

where $X_{i}^{new}(t)$ and $X_{i}(t)$ represent the new and current position vectors of the *i*th particle at the iteration t; $X_{BV,i}$ is the best visible particle vector corresponding to the particle i at the iteration t; $X_{MV,i}(t)$ stands for the mean position vector of all the visible particles associated with the *i*th particle at the iteration t; $rand_{1}$ and $rand_{2}$ are random vectors within the range [0, 1]; β is a random number in the interval [0, 1]; and λ is a number that randomly takes the value of either 1 or 2. 

The second updating operator of the VPSS algorithm also contains two main parts. The first part of this operator tries to update the position of each particle with respect to the differences between its best and worst visible particles. Meanwhile, in the second part, the particle’s position is updated based on the differences between the mean of the visible particles and the worst visible particle. The second updating operator is described as follows:

(10) $$X_{i}^{new}(t)=X_{i}(t)+rand_{1}\cdot \left(X_{BV,i}(t)-\eta \cdot X_{WV,i}(t)\right)+\beta \cdot rand_{2}\cdot \left(X_{MV,i}(t)-\lambda \cdot \;X_{WV,i}(t)\right),$$

where $X_{WV,i}$ denotes the worst visible particle vector corresponding to the particle i at the iteration t; η is a number that randomly takes the value of either 1 or 2; and other parameters are the same as those in the previous operator.

Overall, for each particle at each iteration of the VPSS algorithm, a role switching mechanism is performed to randomly apply one of the two updating operators mentioned above. For this purpose, a random number *r* within the interval [0, 1] is selected. If r≤0.5, the updating operator provided by Equation (9) is adopted, and, if r>0.5, the updating operator offered by Equation (10) is employed.

After the generation of each new solution, its feasibility is evaluated. This implies that, if the solution goes beyond the predefined bounds, it is replaced by the nearest upper or lower bound.

#### 3.2.5. Step 5: Fitness Function Evaluation

In this step, the fitness function value of all the newly generated solutions $fit(X_{i}^{new}),$ i=1,…,Np, is evaluated.

#### 3.2.6. Step 6: Selection

In this step, the newly generated solution $X_{i}^{new}(t)$ is compared with the current solution $X_{i}(t)$. If the new solution $X_{i}^{new}(t)$ results in a better fitness function value, it substitutes the current solution $X_{i}(t)$ in the next iteration of the algorithm. Otherwise, $X_{i}(t)$ is maintained without any change in the population. This procedure can be outlined by the following formula:

(11) $$X_{i}(t+1)=\{\begin{array}{l} \begin{array}{ll} X_{i}^{new}(t), & \mathrm{if}\;fit(X_{i}^{new}(t))\geq fit(X_{i}(t)) \end{array}\\ \begin{array}{ll} X_{i}(t), & \;\;\;\mathrm{if}\;fit(X_{i}^{new}(t))<fit(X_{i}(t)) \end{array} \end{array},$$

where $fit(X_{i}(t))$ and $fit(X_{i}^{new}(t))$ represent the fitness function values of the current solution vector $X_{i}(t)$ and the newly generated solution vector $X_{i}^{new}(t)$, respectively.

#### 3.2.7. Step 7: Termination

The optimization procedure terminates if a stopping criterion is satisfied; otherwise, steps two to six are iteratively carried out. A predefined value for the maximum number of function evaluations can be adopted as the termination criterion. The pseudo-code of the proposed VPSS algorithm is given in Algorithm 1.
**Algorithm 1.** Visible particle series search algorithm   **Begin** 1. Set the population size Np and maximum number of iterations $\;It_{\max}$. 2. Initialize a random population $X_{i},\;i=1,\ldots ,Np,$ using Equation (7). 3. Evaluate the fitness function value of each solution $fit(X_{i})$. 4. Set the current iteration number t=1. 5. **while** ($t\leq It_{\max}$) **do** 6.  Consider the population as a particle series with random arrangement; 7.   Obtain the visible particles associated with each particle using Equation (8); 8.   **for**
i=1 to Np
**do** 9.   Select a random number r from [0, 1]; 10.   **if**
r≤0.5 then 11.    Generate a new solution $X_{i}^{new}(t)$ using Equation (9); 12.   **else** 13.    Generate a new solution $X_{i}^{new}(t)$ using Equation (10); 14.   **end if** 15.   Evaluate the fitness value of the new solution $fit(X_{i}^{new}(t))$; 16.   **if**
$fit(X_{i}^{new}(t))\geq fit(X_{i}(t))$
**then** 17.    $X_{i}(t+1)=X_{i}^{new}(t);$ 18.   **else** 19.    $X_{i}(t+1)=X_{i}(t);$ 20.   **end if** 21.  **end for** 22.   t=t+1; 23. **end while** 24. Return the best solution achieved;    **End**

## 4. Validation of VPSS on Mathematical Benchmark Functions

**Figure 3:**
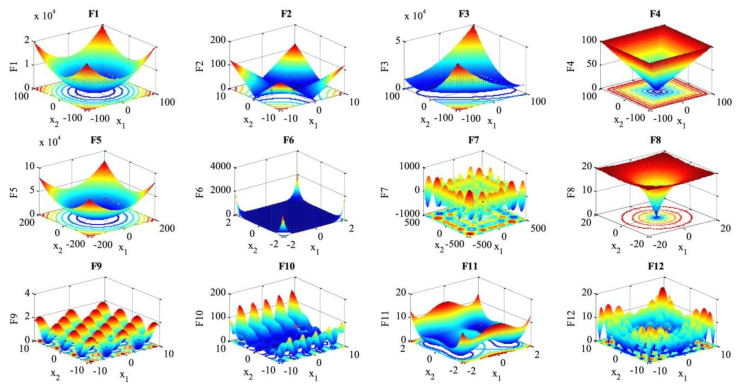
Perspective plots of the mathematical benchmark functions.

In this section, the general performance of the proposed VPSS algorithm is verified through a set of mathematical benchmark functions taken from the literature [61,69,70,71]. The utilized test functions are summarized in Table 1, where *Dim* represents the dimension of the function, and *Range* denotes the search space boundaries. The perspective plots of the test functions considering two variables are also illustrated in Figure 3. The presented benchmark functions can be generally classified into two groups: unimodal functions (*F*_1_–*F*_6_) and multi-modal functions (*F*_7_–*F*_12_). Unimodal functions contain only one global optimum without any local optima. In view of this, these functions are principally applied to evaluate the exploitation capability of optimization algorithms. By contrast, multi-modal functions have several local optima and, hence, are basically employed to examine the exploration capability of optimization algorithms. In addition, both kinds of unimodal and multi-modal functions can be utilized to investigate the convergence behavior of optimization algorithms.

In the case of each mathematical benchmark function, the results attained by VPSS are also compared with those of four well-established optimization algorithms, namely PSO [59], DE [60], GWO [61], and LAPO [62]. For the sake of fairness, the common control parameters are taken the same for all the algorithms. In this regard, the population size is set to 30, and the maximum number of function evaluations is considered to be 15,000. Besides, the values for the specific control parameters of DE and PSO are taken as in [63]. Furthermore, owing to the stochastic nature of the meta-heuristic algorithms, 20 independent runs of all the algorithms are executed for each test function.

Table 2 reports the statistical results obtained by VPSS and the other four algorithms on all the mathematical benchmark functions in terms of the average objective function value (*F_Avg_*) and the corresponding standard deviation value (*F_Std_*). These results reveal that VPSS can achieve the most accurate objective function values among all the compared algorithms in solving all the mathematical benchmark functions. By contrast, LAPO yields the worst results for function *F*_7_, GWO gives the worst results for functions *F*_5_, *F*_10_, and *F*_11_, DE leads to the worst results for functions *F*_1_, *F*_2_, *F*_3_, *F*_4_, *F*_6_, *F*_9_, and *F*_12_, and PSO provides the worst results for function *F*_8_.

According to the results listed in Table 2, the standard deviation values of VPSS are also superior against those of the other optimization algorithms for all the test functions except only function *F*_7_. These results indicate the high-ranking robustness and reliability of the proposed VPSS algorithm.

**Figure 4:**
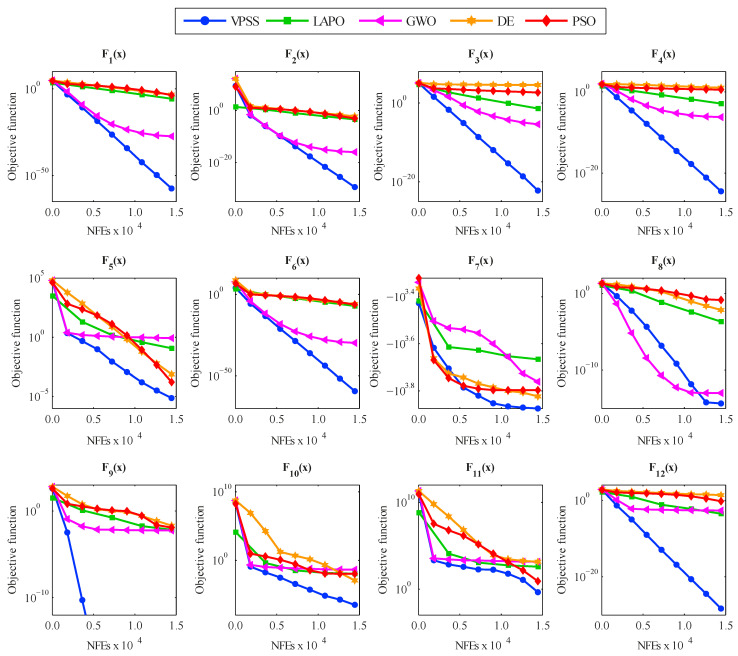
Convergence histories of average objective function values obtained by VPSS, LAPO, GWO, DE, and PSO versus the number of function evaluations (NFEs) for the mathematical benchmark functions.

For further investigation, Figure 4 compares the convergence histories of the average objective function values acquired by VPSS, LAPO, GWO, DE, and PSO for the investigated test functions. The high convergence rate and accuracy of the proposed VPSS algorithm can be inferred from this figure.

Taken together, the optimization results related to the mathematical benchmark functions highlight the promising exploitation and exploration capabilities of the proposed VPSS algorithm.

## 5. Application of VPSS in Structural Damage Identification

In this section, the efficiency of the VPSS algorithm for structural damage identification is assessed by using four numerical examples, comprising a 47-bar planar truss, a 54-bar space truss, a two-bay three-story frame, and a TV tower. Similar to the previous section, the results obtained by VPSS are also compared with those gained by PSO, DE, GWO, and LAPO. In each example, the structural damage identification procedure is implemented by considering both noise-free and noisy conditions. In the noisy condition, the natural frequencies and mode shapes are contaminated by 1% and 10% noise levels, respectively, as in [63]. For all the numerical examples, only the first five natural frequencies and mode shapes are taken into account in formulating the objective function. With regard to the optimization context, the population size is set to be 50, and the maximum number of function evaluations is limited to 20,000 for all the meta-heuristic algorithms. Moreover, ten independent runs of the algorithms are carried out, and the statistical information in terms of the average and standard deviation values are presented.

### 5.1. A 47-Bar Planar Truss

**Figure 5:**
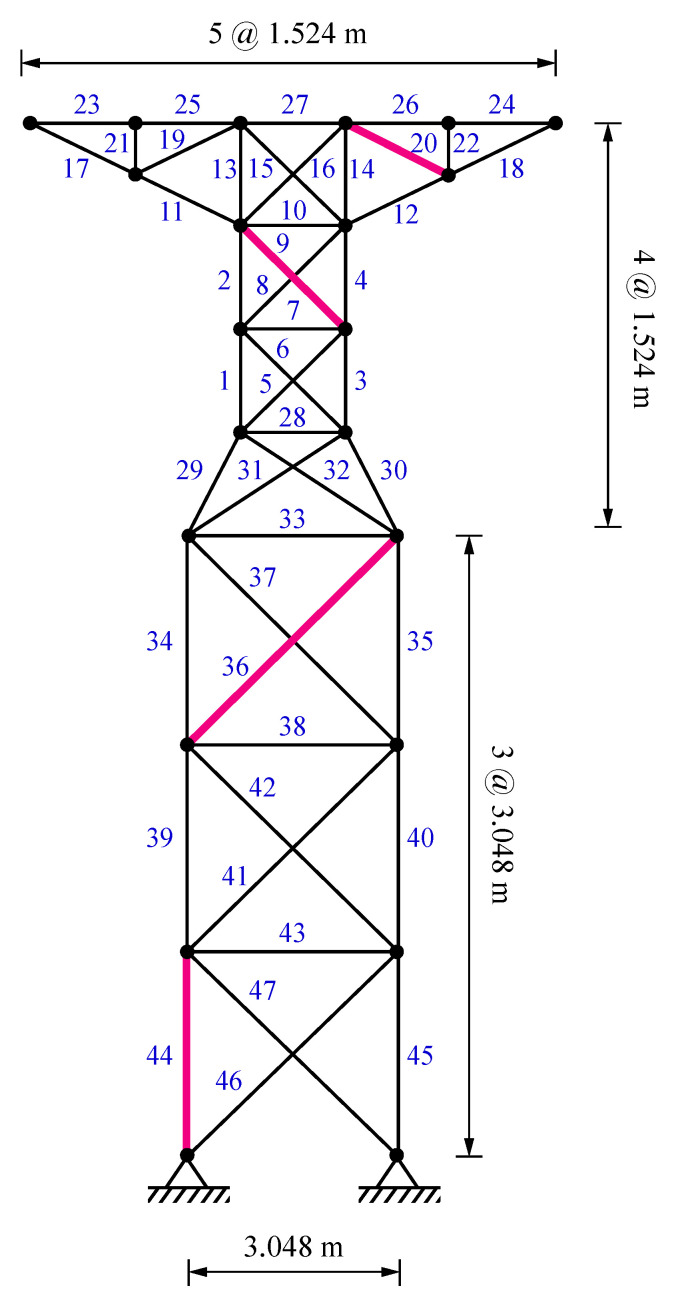
A 47-bar planar truss.

The first damage identification example deals with a 47-bar planar truss structure [43], depicted in Figure 5. The cross-sectional area of elements, modulus of elasticity, and mass density are 0.0025 m^2^, 207 GPa, and 8304 kg/m^3^, respectively. A multi-damage scenario is taken into account by applying damage ratios of 0.30, 0.20, 0.35, and 0.15 to the 9th, 20th, 36th, and 44th members, respectively. The damaged elements are also highlighted in Figure 5.

**Figure 6:**
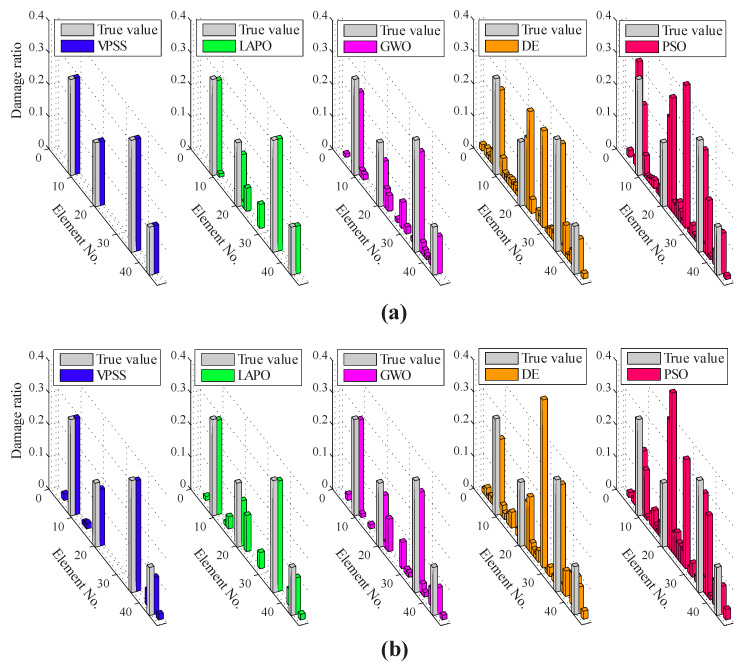
Damage identification results for the 47-bar planar truss under the (**a**) noise-free condition and (**b**) noisy condition.

Figure 6 illustrates the average identification results found by VPSS, LAPO, GWO, DE, and PSO in both the noise-free and noisy conditions. Furthermore, details about the average values, standard deviations, and the maximum false alarm ratio for all the algorithms are reported in Table 3. The identification results clearly indicate the superiority of the proposed VPSS algorithm over the other compared meta-heuristic algorithms with regard to the solution accuracy. Indeed, VPSS can successfully recognize the exact location and extent of the damaged elements in the noise-free condition and further gives highly satisfactory identification outcomes with negligible errors in the noisy condition. By contrast, LAPO, GWO, and especially DE and PSO exhibit significant errors and several misidentifications and fail to perfectly detect the actual damage scenario.

Inspection of Table 3 further indicates that the standard deviations obtained using VPSS are much lower than those of LAPO, GWO, DE, and PSO in both the noise-free and noisy conditions. This signifies the higher reliability of the VPSS algorithm compared with the other meta-heuristic algorithms in addressing the damage identification problem.

**Figure 7:**
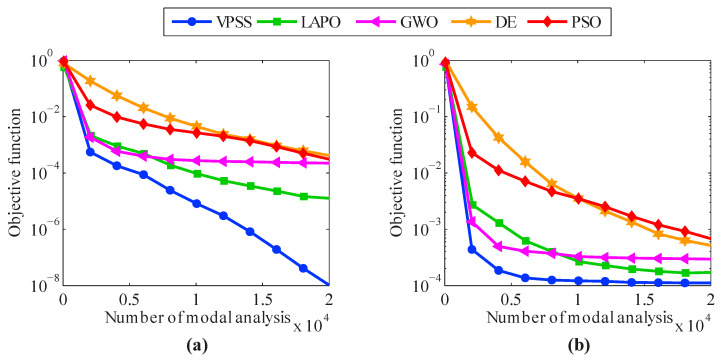
Convergence histories of mean objective function values of VPSS, LAPO, GWO, DE, and PSO for the 47-bar planar truss in the (**a**) noise-free condition and (**b**) noisy condition.

The average convergence histories attained by VPSS, LAPO, GWO, DE, and PSO are compared in Figure 7. Moreover, details of the minimum objective function values achieved by the algorithms are provided in Table 3. As can be observed, the VPSS algorithm significantly outperforms the other compared meta-heuristic algorithms in terms of convergence rate and convergence accuracy.

### 5.2. A 54-Bar Space Truss

**Figure 8:**
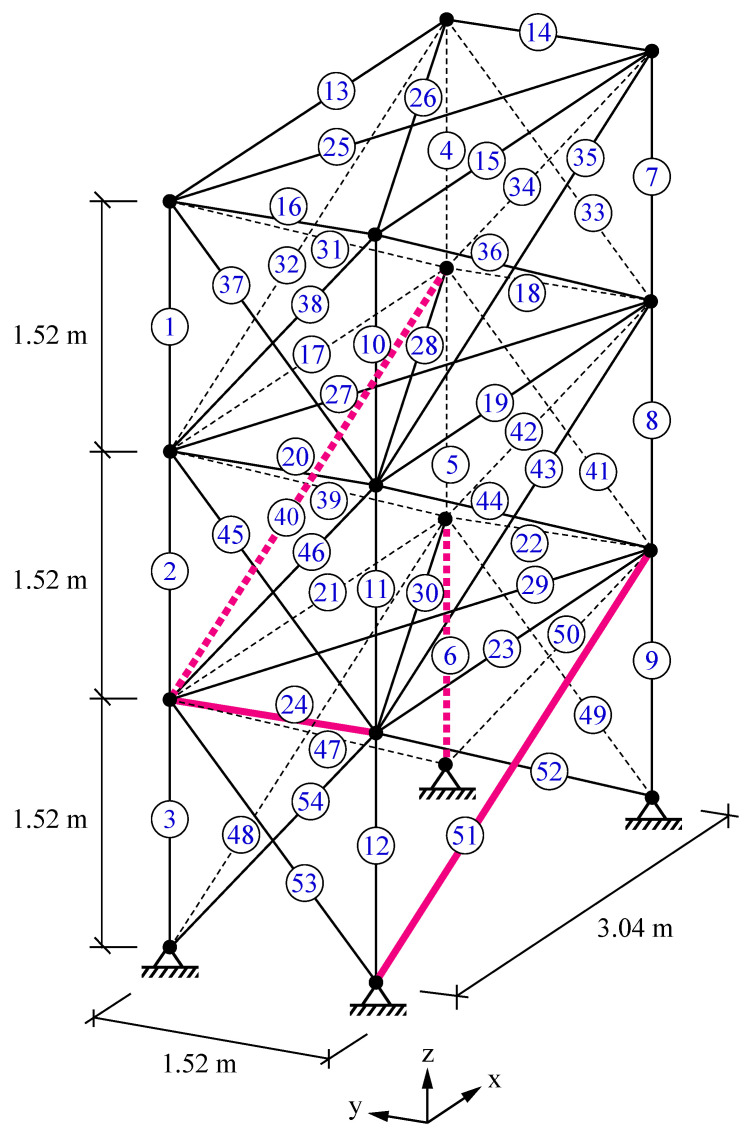
A 54-bar space truss.

A 54-bar space truss [72], as shown in Figure 8, is employed for the second damage identification example. The cross-sectional area of elements, modulus of elasticity, and mass density are 0.0025 m^2^, 68.9 GPa, and 2770 kg/m^3^, respectively. A multi-damage scenario is taken into consideration in which damage ratios of 0.20, 0.15, 0.30, and 0.25 are applied to the 6th, 24th, 40th, and 51st members, respectively. The damaged elements are also indicated in Figure 8.

**Figure 9:**
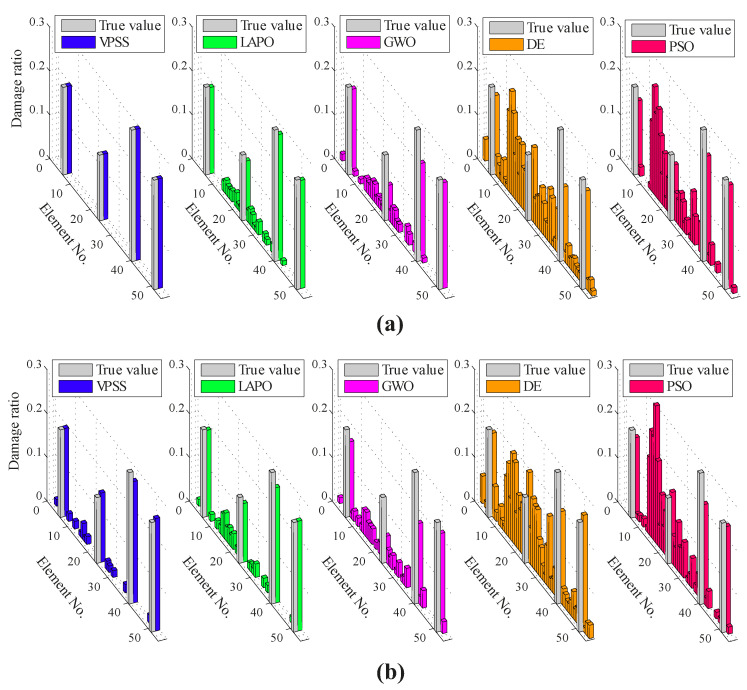
Damage identification results for the 54-bar space truss under the (**a**) noise-free condition and (**b**) noisy condition.

The average identification results acquired by VPSS, LAPO, GWO, DE, and PSO in the noise-free and noisy conditions are shown in Figure 9. In addition, Table 4 provides details about the average values, standard deviations, and the maximum false alarm ratio for all the algorithms. The results demonstrate that VPSS is capable of achieving the most precise identification results among all the compared algorithms. The VPSS algorithm correctly determines the actual damage ratios in the noise-free condition and leads to appropriate estimations with minor errors in the noisy condition. On the other hand, LAPO, despite its relatively acceptable estimations in the noisy condition, carries a number of false alarms in the noise-free condition and, hence, ranks second. GWO, DE, and PSO also produce noticeable errors and misidentifications and, consequently, rank behind VPSS and LAPO.

**Figure 10:**
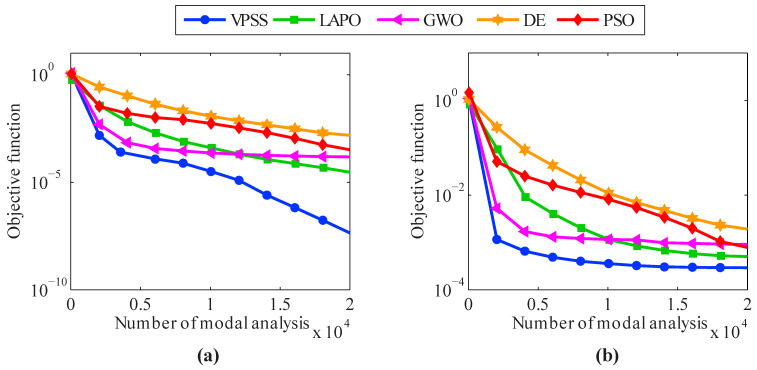
Convergence histories of mean objective function values of VPSS, LAPO, GWO, DE, and PSO for the 54-bar space truss in the (**a**) noise-free condition and (**b**) noisy condition.

From the data in Table 4, it is also apparent that the VPSS algorithm is able to achieve lower standard deviations and, hence, higher robustness compared to the other meta-heuristic algorithms. The average convergence curves of VPSS are compared with those of LAPO, GWO, DE, and PSO in Figure 10. Moreover, details of the minimum objective function values obtained by the algorithms are given in Table 4. These results again confirm the superior convergence performance of the VPSS algorithm compared to the other optimization algorithms.

### 5.3. A Two-Bay Three-Story Frame

**Figure 11:**
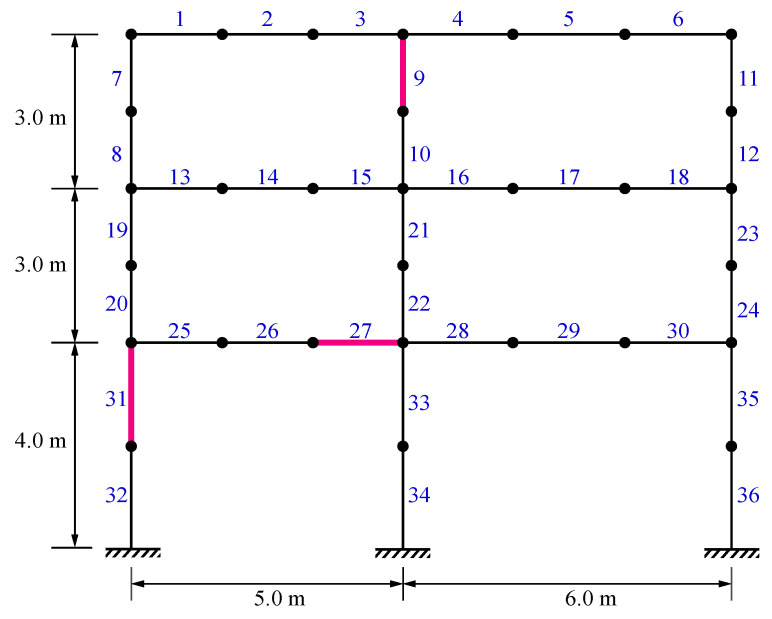
A two-bay three-story frame.

A two-bay three-story frame [73], as illustrated in Figure 11, is considered for the third damage identification example. The cross-sectional areas of beams and columns are 0.0123 m^2^ and 0.0288 m^2^, respectively. The moments of inertia of the beams and columns are 2.219 × 10^−4^ m^4^ and 5.744 × 10^−4^ m^4^, respectively. Moreover, the elasticity modulus and the mass density of all the elements are 207 GPa and 7780 kg/m^3^, respectively. A multi-damage scenario is considered by applying damage ratios of 0.25, 0.35, and 0.15 to the 9th, 27th, and 31st members, respectively. The damaged elements are also highlighted in Figure 11.

**Figure 12:**
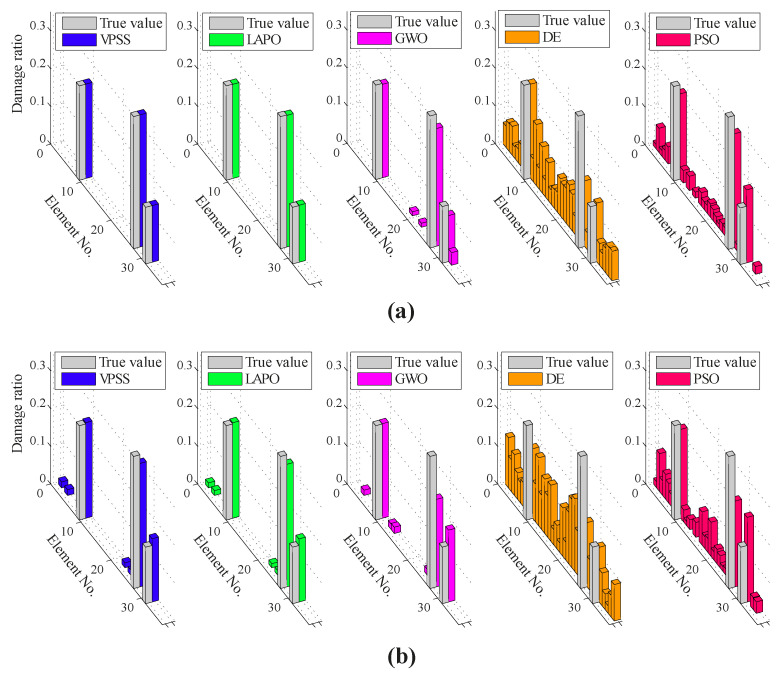
Damage identification results for the two-bay three-story frame under the (**a**) noise-free condition and (**b**) noisy condition.

Figure 12 demonstrates the average identification results achieved by VPSS, LAPO, GWO, DE, and PSO in both the noise-free and noisy conditions. Additionally, Table 5 summarizes details about the average values, standard deviations, and the maximum false alarm ratio for all the algorithms. As can be observed, VPSS and LAPO possess approximately the same level of accuracy and outperform the other optimization algorithms.

It is further evident from Table 5 that VPSS can lead to lower standard deviations compared with the other algorithms in both the noise-free and noisy conditions.

**Figure 13:**
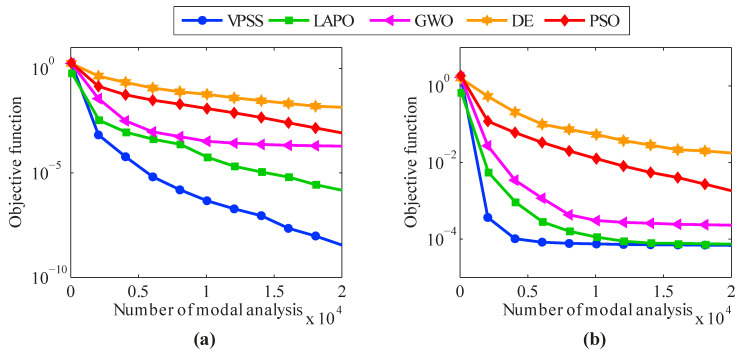
Convergence histories of mean objective function values of VPSS, LAPO, GWO, DE, and PSO for the two-bay three-story frame in the (**a**) noise-free condition and (**b**) noisy condition.

The average convergence histories attained by VPSS, LAPO, GWO, DE, and PSO are illustrated in Figure 13. Moreover, details of the minimum objective function values gained by the algorithms are presented in Table 5. As shown, all the compared algorithms in both the noise-free and noisy conditions are inferior to the VPSS algorithm with regard to convergence speed and convergence accuracy except for LAPO in the noisy condition, which can approximately reach the same level of accuracy as the VPSS algorithm.

### 5.4. The Canton Tower

**Figure 14:**
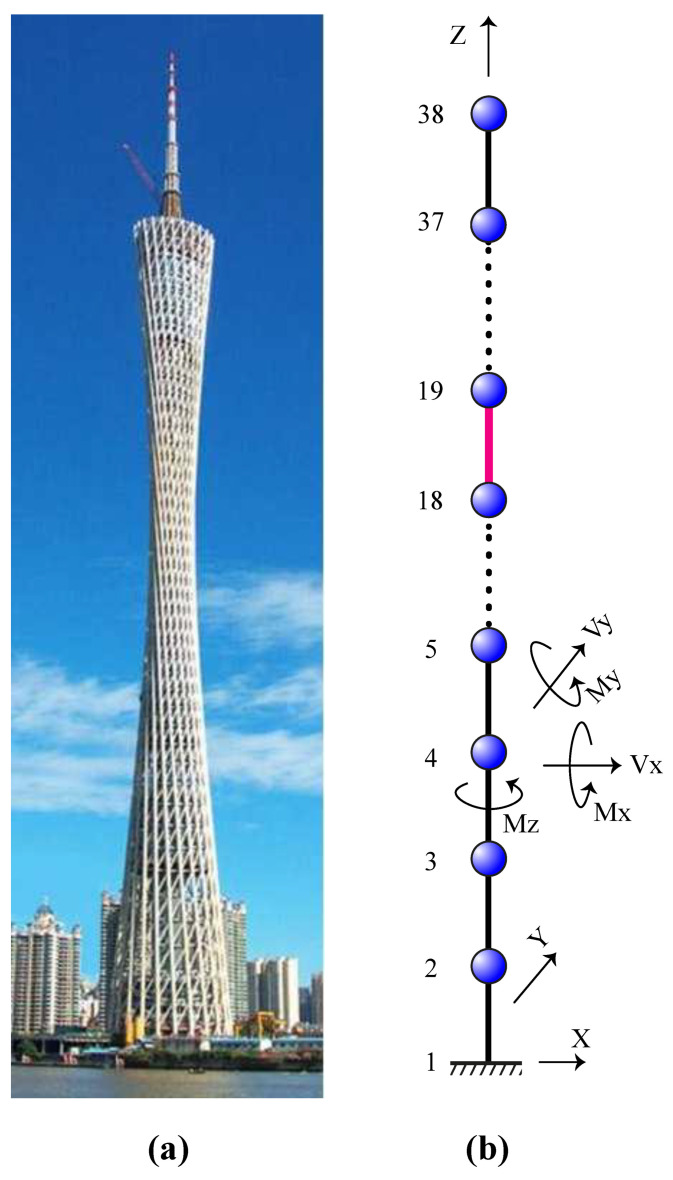
The Canton Tower: (**a**) overview (**b**) reduced-order finite element model.

For the last damage identification example, the Canton Tower shown in Figure 14a is considered. This structure has a total height of 610 m, which makes it the second tallest TV tower in the world. The full-order finite element model of Canton Tower comprises 122,476 elements, 84,370 nodes, and 505,164 degrees-of-freedom (DOFs) [74]. Due to the high complexity of this model, an equivalent reduced-order finite element model is generated in accordance with [74,75] to achieve the damage identification task. As shown in Figure 14b, the reduced model represents a three-dimensional cantilever beam containing 37 elements and 38 nodes. At each node, five DOFs, including two translational displacements in the x and y directions and three rotational displacements about the x, y, and z directions, are defined. Therefore, there are 10 DOFs per element, and the whole structural model possesses a total of 185 DOFs. For this example, a single damage scenario is simulated by considering the damage ratio of 0.20 to the 18th member, which is highlighted in Figure 14b. It is also assumed that the limited mode shape data obtained from nodes 2, 7, 12, 17, 22, 27, 32, and 37 are utilized for damage identification.

**Figure 15:**
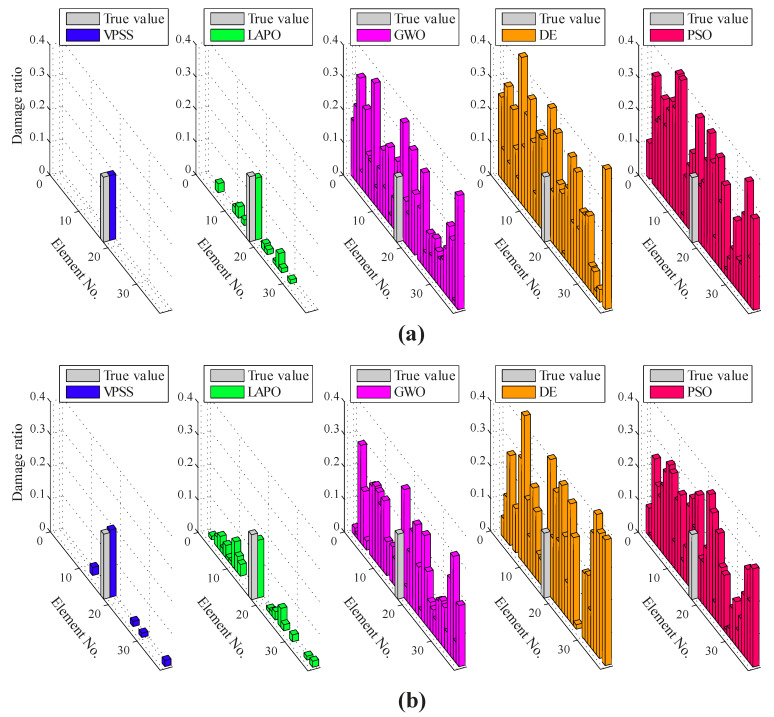
Damage identification results for the Canton Tower under the (**a**) noise-free condition and (**b**) noisy condition.

Figure 15 illustrates the average identification results found by VPSS, LAPO, GWO, DE, and PSO in both the noise-free and noisy conditions. Furthermore, details about the average values, standard deviations, and the maximum false alarm ratio for all the algorithms are reported in Table 6. It is evident from the results that the VPSS algorithm yields the most promising identification results among all the comparative algorithms. In fact, the VPSS algorithm can precisely assess the actual damage state of the structure in the noise-free condition and still provide acceptable identification outcomes with marginal errors in the noisy condition. Meanwhile, the LAPO algorithm gives approximate results with overall accuracy lower than VPSS and occupies the second rank. However, GWO, DE, and PSO carry several misidentifications and cannot identify the damage scenario at all.

According to Table 6, it is also observed that the standard deviations gained by the VPSS algorithm are much lower than those reported for the other algorithms in both the noise-free and noisy conditions.

**Figure 16:**
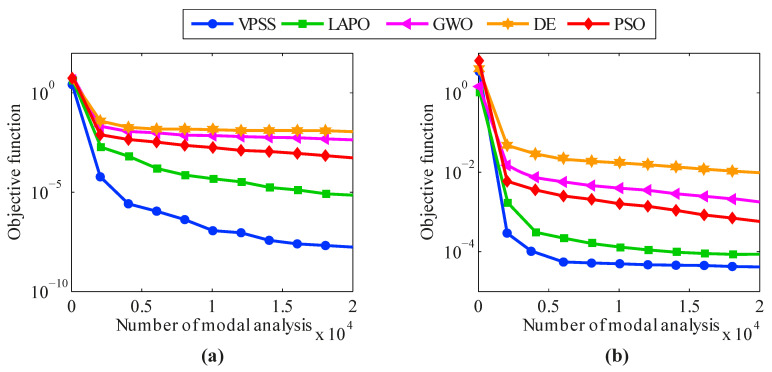
Convergence histories of mean objective function values of VPSS, LAPO, GWO, DE, and PSO for the Canton Tower in the (**a**) noise-free condition and (**b**) noisy condition.

The average convergence histories achieved by VPSS, LAPO, GWO, DE, and PSO are illustrated in Figure 16. Moreover, details about the average values, standard deviations, and the maximum false alarm ratio for all the algorithms are given in Table 6. As can be observed, the VPSS algorithm performs considerably better than the other compared meta-heuristic algorithms with respect to the convergence rate and convergence precision.

## 6. Conclusions

This paper proposed a new meta-heuristic optimization algorithm named visible particle series search (VPSS) for structural damage identification. The VPSS algorithm was inspired by the visibility graph technique, which is mainly applied in the area of time-series analysis to characterize the intrinsic features of a time-series by mapping it into a graph network. In the VPSS algorithm, the population of candidate solutions is viewed as a particle series and then is transformed into a visibility graph representation. Any two particles are visible to each other in the graph if there is a straight line connecting them without intersecting any intermediate particles. The VPSS algorithm updates the position of the particles in the search space by taking advantage of information derived from the visible particles. This algorithm requires only the common controlling parameters and is free from any algorithm-specific parameter.

In order to verify the exploitation and exploration capabilities of VPSS, a general test was first carried out on 12 mathematical benchmark functions, comprising unimodal and multi-modal functions. Next, the efficiency of this algorithm for identifying structural damage was investigated by using four numerical examples, including a 47-bar planar truss, a 54-bar space truss, a two-bay three-story frame, and a TV tower, under both noise-free and noisy conditions. For each optimization problem, comparisons were also performed between the results of VPSS and those gained by four other well-known meta-heuristic algorithms, namely particle swarm optimization (PSO), differential evolution (DE), grey wolf optimization (GWO), and lightning attachment procedure optimization (LAPO). The results of the mathematical functions and damage identification problems reveal that VPSS outperforms the other meta-heuristic algorithms in terms of accuracy, robustness, and convergence speed.

## Figures and Tables

**Table 1:** Mathematical benchmark functions.

No.	Name	Formula	Dim	Range
$F_{1}$	Sphere	$F_{1}(x)=\sum_{i=1}^{D}x_{i}^{2}$	30	[−100, 100]
$F_{2}$	Schwefel 2.22	$F_{2}(x)=\sum_{i=1}^{D}\left|x_{i}\right|+\prod_{i=1}^{n}\left|x_{i}\right|$	30	[−10, 10]
$F_{3}$	Schwefel 1.2	$F_{3}(x)=\sum_{i=1}^{D}{\left(\sum_{j=1}^{i}x_{i}\right)}^{2}$	30	[−100, 100]
$F_{4}$	Schwefel 2.21	$F_{4}(x)=\max_{i}\left\{\left|x_{i}\right|,\;1\leq i\leq D\right\}$	30	[−100, 100]
$F_{5}$	Step 2	$F_{5}(x)={\sum_{i=1}^{D}\left(\left\lfloor x_{i}+0.5\right\rfloor \right)}^{2}$	30	[−100, 100]
$F_{6}$	Brown	$F_{6}(x)={\sum_{i=1}^{D-1}\left(x_{i}\right)}^{\left(x_{i+1}^{2}+1\right)}+{\left(x_{i+1}^{2}\right)}^{\left(x_{i}^{2}+1\right)}$	30	[−1, 4]
$F_{7}$	Schwefel 2.26	$F_{7}(x)=\sum_{i=1}^{D}-x_{i}\sin(\sqrt{\left|x_{i}\right|})$	30	[−500, 500]
$F_{8}$	Ackley	$F_{8}(x)=-20e^{-0.02\sqrt{D^{-1}\sum_{i=1}^{D}x_{i}^{2}}}-e^{D^{-1}\sum_{i=1}^{D}\cos(2\pi x_{i})}+20+e$	30	[−32, 32]
$F_{9}$	Griewank	$F_{9}(x)=\frac{1}{400}\sum_{i=1}^{D}\left(x_{i}^{2}\right)-\left(\prod_{i=1}^{D}\cos\left(\frac{x_{i}}{\sqrt{i}}\right)\right)+1$	30	[−600, 600]
$F_{10}$	Penalized	$\begin{array}{ll} F_{10}(x)= & \frac{\pi }{D}\{10\sin^{2}(\pi y_{i})+\sum_{i=1}^{D-1}{\left(y_{i}-1\right)}^{2}\left[1+10\sin^{2}(\pi y_{i+1})\right]\\ & +{\left(y_{D}-1\right)}^{2}\}+\sum_{i=1}^{D}u(x_{i},10,100,4),\\ & y_{i}=1+\frac{1}{4}(x_{i}+1),\\ & u(x_{i},a,k,m)=\{\begin{array}{cc} k{\left(x_{i}-a\right)}^{m}, & x_{i}>a,\\ 0, & -a\leq x_{i}\leq a,\\ k{\left(-x_{i}-a\right)}^{m}, & x_{i}<-a. \end{array} \end{array}$	30	[−50, 50]
$F_{11}$	Qing	$F_{11}(x)=\sum_{i=1}^{D}{\left(x_{i}^{2}-i\right)}^{2}$	30	[−500, 500]
$F_{12}$	Alpine 1	$F_{12}(x)=\sum_{i=1}^{D}\left|x_{i}\sin(x_{i})+0.1x_{i}\right|$	30	[−100, 100]

**Table 2:** Statistical results for the mathematical benchmark functions.

No.	VPSS		LAPO		GWO		DE		PSO	
	$F_{Avg}$	$F_{Std}$	$F_{Avg}$	$F_{Std}$	$F_{Avg}$	$F_{Std}$	$F_{Avg}$	$F_{Std}$	$F_{Avg}$	$F_{Std}$
*F*_1_	1.1 × 10^−60^	2.1 × 10^−60^	8.3 × 10^−7^	8.1 × 10^−7^	3.7 × 10^−28^	5.2 × 10^−28^	2.6 × 10^−4^	9.8 × 10^−5^	7.1 × 10^−5^	6.8 × 10^−5^
*F*_2_	2.2 × 10^−31^	1.8 × 10^−31^	2.9 × 10^−4^	2.9 × 10^−4^	9.7 × 10^−17^	3.9 × 10^−17^	3.9 × 10^−3^	1.0 × 10^−3^	4.5 × 10^−4^	2.4 × 10^−4^
*F*_3_	8.1 × 10^−24^	1.9 × 10^−23^	2.9 × 10^−2^	4.2 × 10^−2^	3.9 × 10^−6^	7.2 × 10^−6^	37450	5799.2	427.34	191.25
*F*_4_	3.4 × 10^−26^	2.4 × 10^−26^	8.5 × 10^−4^	4.7 × 10^−4^	5.7 × 10^−7^	5.1 × 10^−7^	9.4889	2.0314	3.2985	7.6 × 10^−1^
*F*_5_	4.8 × 10^−6^	7.2 × 10^−6^	1.0 × 10^−1^	1.2 × 10^−1^	8.6 × 10^−1^	4.6 × 10^−1^	3.4 × 10^−4^	1.3 × 10^−4^	3.6 × 10^−5^	5.8 × 10^−5^
*F*_6_	1.6 × 10^−62^	3.2 × 10^−62^	3.1 × 10^−8^	4.0 × 10^−8^	1.3 × 10^−30^	1.5 × 10^−30^	5.6 × 10^−7^	2.5 × 10^−7^	3.2 × 10^−7^	6.0 × 10^−7^
*F*_7_	−7508.4	1262.5	−4639.1	312.88	−5822.0	995.73	−6706.5	420.66	−6272.1	543.41
*F*_8_	4.4 × 10^−15^	0.0000	1.4 × 10^−4^	1.4 × 10^−4^	1.0 × 10^−13^	9.5 × 10^−15^	5.1 × 10^−3^	2.2 × 10^−3^	1.3 × 10^−1^	4.2 × 10^−1^
*F*_9_	0.0000	0.0000	6.3 × 10^−3^	1.3 × 10^−2^	5.7 × 10^−3^	1.3 × 10^−2^	1.8 × 10^−2^	5.4 × 10^−2^	1.2 × 10^−2^	1.3 × 10^−2^
*F*_10_	1.9 × 10^−7^	1.8 × 10^−7^	1.1 × 10^−2^	3.2 × 10^−2^	4.1 × 10^−2^	2.1 × 10^−2^	5.9 × 10^−4^	6.2 × 10^−4^	1.0 × 10^−2^	3.2 × 10-^02^
*F*_11_	1.6 × 10^−1^	2.9 × 10^−1^	359.96	723.34	1.5 × 10^+03^	534.71	1320.8	222.23	2.6347	4.6566
*F*_12_	3.5 × 10^−30^	5.5 × 10^−30^	2.1 × 10^−4^	2.0 × 10^−4^	1.9 × 10^−3^	1.5 × 10^−3^	20.253	3.3790	3.4 × 10^−1^	4.1 × 10^−1^

**Table 3:** Statistical results of the damage identification for the 47-bar planar truss.

Condition	Method	Damage Variables								$x_{MFA}$	$F_{\min}$
		$x_{9}$		$x_{20}$		$x_{36}$		$x_{44}$			
		Avg.	Sdt.	Avg.	Sdt.	Avg.	Sdt.	Avg.	Sdt.		
Noise-free	VPSS	0.300	3.7 × 10^−4^	0.200	5.1 × 10^−4^	0.350	2.7 × 10^−4^	0.150	8.7 × 10^−5^	0.000	1.0 × 10^−8^
	LAPO	0.293	5.1 × 10^−3^	0.159	5.4 × 10^−2^	0.350	1.6 × 10^−3^	0.148	2.3 × 10^−3^	0.074	1.3 × 10^−5^
	GWO	0.256	8.9 × 10^−2^	0.139	8.9 × 10^−2^	0.309	1.1 × 10^−1^	0.116	6.1 × 10^−2^	0.081	2.2 × 10^−4^
	DE	0.260	2.7 × 10^−2^	0.142	8.5 × 10^−2^	0.332	3.2 × 10^−2^	0.106	2.8 × 10^−2^	0.301	4.2 × 10^−4^
	PSO	0.216	5.3 × 10^−2^	0.181	6.0 × 10^−2^	0.315	2.4 × 10^−2^	0.127	1.4 × 10^−2^	0.420	3.1 × 10^−4^
Noisy	VPSS	0.300	2.0 × 10^−3^	0.176	1.8 × 10^−3^	0.345	1.6 × 10^−3^	0.115	1.7 × 10^−3^	0.049	1.1 × 10^−4^
	LAPO	0.294	5.9 × 10^−3^	0.141	7.1 × 10^−2^	0.345	3.6 × 10^−3^	0.114	1.9 × 10^−3^	0.112	1.7 × 10^−4^
	GWO	0.295	9.4 × 10^−3^	0.158	6.2 × 10^−2^	0.310	1.1 × 10^−1^	0.083	5.5 × 10^−2^	0.101	2.9 × 10^−4^
	DE	0.232	2.9 × 10^−2^	0.093	7.4 × 10^−2^	0.331	4.3 × 10^−2^	0.083	2.4 × 10^−2^	0.560	5.1 × 10^−4^
	PSO	0.198	4.6 × 10^−2^	0.171	6.9 × 10^−2^	0.306	2.7 × 10^−2^	0.087	2.7 × 10^−2^	0.610	6.8 × 10^−4^

Note: Avg. = average; Std. = standard deviation; $x_{MFA}$ *=* maximum false alarm ratio; *F*_min_ = minimum objective function value achieved.

**Table 4:** Statistical results of the damage identification for the 54-bar space truss.

Condition	Method	Damage Variables								$x_{MFA}$	$F_{\min}$
		*x*_6_		*x*_24_		*x*_40_		*x*_51_			
		Avg.	Sdt.	Avg.	Sdt.	Avg.	Sdt.	Avg.	Sdt.		
Noise-free	VPSS	0.200	2.4 × 10^−4^	0.150	5.4 × 10^−4^	0.300	5.8 × 10^−4^	0.250	1.9 × 10^−4^	0.000	4.3 × 10^−8^
	LAPO	0.197	5.5 × 10^−3^	0.134	1.7 × 10^−2^	0.287	1.0 × 10^−2^	0.246	4.0 × 10^−3^	0.035	2.9 × 10^−5^
	GWO	0.193	6.9 × 10^−3^	0.078	6.4 × 10^−2^	0.221	1.2 × 10^−1^	0.240	5.4 × 10^−3^	0.041	1.5 × 10^−4^
	DE	0.178	1.9 × 10^−2^	0.094	1.0 × 10^−1^	0.168	7.9 × 10^−2^	0.223	1.7 × 10^−2^	0.233	1.5 × 10^−3^
	PSO	0.167	1.1 × 10^−2^	0.125	1.9 × 10^−2^	0.238	3.2 × 10^−2^	0.236	8.2 × 10^−3^	0.246	2.4 × 10^−4^
Noisy	VPSS	0.200	1.1 × 10^−3^	0.158	9.6 × 10^−3^	0.277	1.8 × 10^−3^	0.256	4.0 × 10^−3^	0.035	2.9 × 10^−4^
	LAPO	0.196	5.2 × 10^−3^	0.134	4.5 × 10^−2^	0.263	2.2 × 10^−2^	0.250	9.4 × 10^−3^	0.059	5.0 × 10^−4^
	GWO	0.170	6.2 × 10^−2^	0.059	8.1 × 10^−2^	0.181	1.2 × 10^−1^	0.223	7.8 × 10^−2^	0.054	9.1 × 10^−4^
	DE	0.189	2.8 × 10^−2^	0.204	9.9 × 10^−2^	0.208	8.1 × 10^−2^	0.264	3.1 × 10^−2^	0.200	1.9 × 10^−3^
	PSO	0.181	1.2 × 10^−2^	0.160	1.9 × 10^−2^	0.226	3.5 × 10^−2^	0.240	1.2 × 10^−2^	0.320	8.6 × 10^−4^

Note: Avg. = average; Std. = standard deviation; $x_{MFA}$ *=* maximum false alarm ratio; *F*_min_ = minimum objective function value achieved.

**Table 5:** Statistical results of the damage identification for the two-bay three-story frame.

Condition	Method	Damage Variables						$x_{MFA}$	$F_{\min}$
		$x_{9}$		$x_{27}$		$x_{31}$			
		Avg	Std	Avg	Std	Avg	Std		
Noise-free	VPSS	0.250	7.1 × 10^−5^	0.350	8.3 × 10^−5^	0.150	1.1 × 10^−4^	0.000	3.5 × 10^−9^
	LAPO	0.249	7.0 × 10^−4^	0.349	1.6 × 10^−3^	0.151	1.4 × 10^−3^	0.000	1.5 × 10^−6^
	GWO	0.247	6.2 × 10^−3^	0.312	1.4 × 10^−1^	0.121	9.0 × 10^−2^	0.033	1.9 × 10^−4^
	DE	0.248	9.6 × 10^−2^	0.174	1.8 × 10^−1^	0.155	1.3 × 10^−1^	0.161	1.3 × 10^−2^
	PSO	0.226	2.1 × 10^−2^	0.302	3.2 × 10^−2^	0.193	2.8 × 10^−2^	0.064	8.2 × 10^−4^
Noisy	VPSS	0.253	1.2 × 10^−3^	0.327	3.9 × 10^−3^	0.168	1.6 × 10^−3^	0.012	6.8 × 10^−5^
	LAPO	0.253	3.6 × 10^−3^	0.325	5.5 × 10^−3^	0.167	6.7 × 10^−3^	0.015	7.0 × 10^−5^
	GWO	0.250	4.4 × 10^−3^	0.232	1.5 × 10^−1^	0.190	3.9 × 10^−2^	0.016	2.3 × 10^−4^
	DE	0.184	1.2 × 10^−1^	0.170	1.2 × 10^−1^	0.145	1.1 × 10^−1^	0.180	1.7 × 10^−2^
	PSO	0.236	8.9 × 10^−3^	0.227	5.9 × 10^−2^	0.224	3.0 × 10^−2^	0.100	1.8 × 10^−3^

Note: Avg. = average; Std. = standard deviation; $x_{MFA}$ *=* maximum false alarm ratio; *F*_min_ = minimum objective function value achieved.

**Table 6:** Statistical results of the damage identification for the Canton Tower.

Condition	Method	Damage Variables		$x_{MFA}$	$F_{\min}$
		*x*_18_			
		Avg.	Sdt.		
Noise-free	VPSS	0.200	8.1 × 10^−4^	0.000	1.7 × 10^−8^
	LAPO	0.190	5.3 × 10^−3^	0.047	7.2 × 10^−6^
	GWO	0.362	5.8 × 10^−2^	0.372	4.3 × 10^−3^
	DE	0.406	2.0 × 10^−2^	0425	1.1 × 10^−2^
	PSO	0.378	9.7 × 10^−3^	0.410	5.4 × 10^−4^
Noisy	VPSS	0.207	7.3 × 10^−3^	0.024	4.1 × 10^−5^
	LAPO	0.178	9.9 × 10^−3^	0.082	8.7 × 10^−5^
	GWO	0.332	7.9 × 10^−2^	0.300	1.6 × 10^−3^
	DE	0.421	4.9 × 10^−2^	0.430	9.8 × 10^−3^
	PSO	0.315	3.7 × 10^−2^	0.362	5.7 × 10^−4^

Note: Avg. = average; Std. = standard deviation; $x_{MFA}$ *=* maximum false alarm ratio; *F*_min_ = minimum objective function value achieved.

## Data Availability

This research received no data availability statement.
